# Distinct influences of tandem repeats and retrotransposons on CENH3 nucleosome positioning

**DOI:** 10.1186/1756-8935-4-3

**Published:** 2011-02-25

**Authors:** Jonathan I Gent, Kevin L Schneider, Christopher N Topp, Carmen Rodriguez, Gernot G Presting, R Kelly Dawe

**Affiliations:** 1 Department of Plant Biology, University of Georgia, Athens, Georgia, USA; 2 Department of Genetics, University of Georgia, Athens, Georgia, USA; 3 Molecular Biosciences and Bioengineering, University of Hawaii, Honolulu, Hawaii, USA

## Abstract

**Background:**

Unique structural characteristics of centromere chromatin enable it to support assembly of the kinetochore and its associated tensions. The histone H3 variant CENH3 (centromeric histone H3) is viewed as the key element of centromere chromatin and its interaction with centromere DNA is epigenetic in that its localization to centromeres is not sequence-dependent.

**Results:**

In order to investigate what influence the DNA sequence exerts on CENH3 chromatin structure, we examined CENH3 nucleosome footprints on maize centromere DNA. We found a predominant average nucleosome spacing pattern of roughly 190-bp intervals, which was also the dominant arrangement for nucleosomes genome-wide. For CENH3-containing nucleosomes, distinct modes of nucleosome positioning were evident within that general spacing constraint. Over arrays of the major ~156-bp centromeric satellite sequence (tandem repeat) *CentC*, nucleosomes were not positioned in register with *CentC *monomers but in conformity with a striking ~10-bp periodicity of AA/TT dimers within the sequence. In contrast, nucleosomes on a class of centromeric retrotransposon (*CRM2*) lacked a detectable AA/TT periodicity but exhibited tightly phased positioning.

**Conclusions:**

These data support a model in which general chromatin factors independent of both DNA sequence and CENH3 enforce roughly uniform centromeric nucleosome spacing while allowing flexibility in the mode in which nucleosomes are positioned. In the case of tandem repeat DNA, the natural bending effects related to AA/TT periodicity produce an energetically-favourable arrangement consistent with conformationally rigid nucleosomes and stable chromatin at centromeres.

## Background

Centromeres are the regions of chromosomes where kinetochores form and microtubules attach to guide chromosomes to opposite poles during cell division. A wide variety of centromere forms are observed in the diverse cellular contexts of different organisms, from the small point centromeres of *Saccharomyces cerevisiae *to the diffuse, holocentric centromeres of *Caenorhabditis elegans*. Even within a single individual, centromeres must meet the distinct requirements of chromosome dynamics in both meiotic and mitotic cell division. From the outset, one would guess that centromere DNA elements would specify binding sites for structural and regulatory proteins. Indeed, specific, functionally important DNA sequences have been identified, such as the CENP-B (centromere protein B) box site in vertebrates [1-3] and the CP1 binding site in *S. cerevisiae *[4,5]. Intriguingly, however, multiple lines of evidence indicate that core kinetochore proteins can have a large degree of sequence independence (for recent review, see [6]). Foremost among these arguments is the unexpectedly low level of DNA conservation in centromeres. General structural features are common, most notably large arrays of repeated units (called centromere tandem repeats or satellite DNA) and retrotransposons. In addition, the core centromeres, where the kinetochores form, are often flanked by pericentromeres that may contain similar repetitive DNA. Core centromeres are often megabases in length, as are the pericentromeres.

The defining distinction between functional core centromeres and all other chromosomal regions lies in its chromatin composition: specifically, histone H3 of canonical nucleosomes is replaced by a variant called centromeric histone H3 (CENH3; CENP-A in mammals). The precise structure of CENH3 nucleosomes and higher dimensional chromatin is a matter of much investigation and may differ considerably in different cellular contexts (for recent review, see [6]). Given the ambiguous role of DNA sequence in specifying centromere identity and function, it is of considerable importance to understand what contribution DNA sequence makes to chromatin structure. A central question related to both CENH3 and canonical histone H3 nucleosomes concerns interactions between histones and DNA. Both *in vitro *and *in vivo *studies have demonstrated preferences for nucleosomes to bind DNA containing an approximate 10-base periodicity of AA or TT dinucleotides [7-9]. This nucleotide distribution, along with others, favours the tight bending and twisting of the DNA double helix required for wrapping around a histone octamer [10,11]. A propensity for tight bending is a conserved feature of satellite DNA, and it is thought to contribute to the structural stability and compaction of centromere chromatin [12-15]. In addition, satellite DNA in humans, in conjunction with CENP-B, favours a phased arrangement of nucleosomes, where nucleosomes often reproducibly adopt the same positions on the DNA (relative to satellite dimers) [1,3,16]. Nucleosomes of African green monkeys are also preferentially positioned but do not depend on the canonical CENP-B binding site, suggesting that histone-DNA sequence affinities or other proteins may influence the placement of nucleosomes [17-22]. In these species the satellite DNA unit is about 171 bp - just long enough to accommodate a single nucleosome core (histone octomer) and linker (the DNA between nucleosomes cores, bound by histone H1). However, in other species such a relationship is probably impossible because the satellite unit is too small. For example, in mouse the basic satellite unit is 120 bp, in sugarcane it is 140 bp, and in soybean it is two separate units of 92 and 411-bp [23,24].

Factors such as transcription are also known to play important roles in nucleosome positioning [25-27]. Centromere elements such as satellites are thought to be transcriptionally active, although it is unclear how centromeric transcripts are initiated (for a recent review, see [28]). Questions related to transcription and nucleosome positioning are of general interest in centromere regions because of the potential contribution to both kinetochore assembly and large-scale chromosome dynamics. In addition, the contribution of other centromere DNA elements such as retrotransposons to centromere chromatin structure has received much less attention than satellites but is, nevertheless, of importance given the abundance of these elements in centromeres.

The availability of deep sequencing technology prompted us to explore the relation between DNA sequence and *in vivo *CENH3 chromatin structure on centromeres in maize (*Zea mays*). Through analysis of the CENH3 nucleosome core footprints at single-molecule resolution provided by 454 sequencing and through other approaches, we found a tendency for nucleosome spacing intervals to average about 190 bp, which was maintained across the three major DNA components of maize centromeres. These three different DNA elements, however, were sharply distinguished from each other by modes of nucleosome positioning. On arrays of the 156-bp satellite (tandem repeat) *CentC *we found no evidence for nucleosome phasing but, instead, found that the nucleosome positioning was in conformity with an underlying ~10-bp periodity of AA/TT dimers. Nucleosomes on the two centromeric retrotransposons *CRM1 *and *CRM2 *lacked a detectable correlation with AA/TT periodicity but *CRM2 *showed strong nucleosome phasing while *CRM1 *did not. We conclude from these data that DNA elements within centromeres can have distinct effects on chromatin structure in terms of nucleosome positioning (and hence conformational rigidity of the local chromatin).

## Results and discussion

### Derivation and validation of CENH3 nucleosome core sequences

A dataset of DNA sequences associated with maize CENH3 chromatin was described previously as a resource for defining functional centromere regions (core centromeres) [29,30]; GenBank Sequence Read Archive SRA009397). This dataset also provides a unique opportunity to characterize CENH3 nucleosome footprints at the nucleotide level due to both the method of preparing DNA fragments and the sequencing technology used. In particular, digestion of isolated chromatin with micrococcal nuclease eliminated unprotected DNA while preserving the stretches that were tightly bound by protein. Chromatin immunoprecipitation (ChIP) with antibodies against CENH3 then enriched for DNA/protein complexes containing CENH3. 454 sequencing with read lengths of up to 300 bases allowed for precise mapping of DNA fragment endpoints. (The normal steps to remove small fragments were omitted in order to avoid biasing towards large DNA fragments). In order to define the DNA fragments at high resolution, we first filtered through the resulting 149,756 raw reads to remove the ones where the 3' end of the DNA fragment could not be identified due to low sequence quality or fragment lengths in excess of the read length capability. We aligned the resulting set of 110,678 reads to the maize genome to filter out junk DNA fragments (for example, artefacts of library preparation and contamination by non-maize DNA), to produce a set of 105,284 reads corresponding to putative CENH3 nucleosome cores.

**Figure 1:**
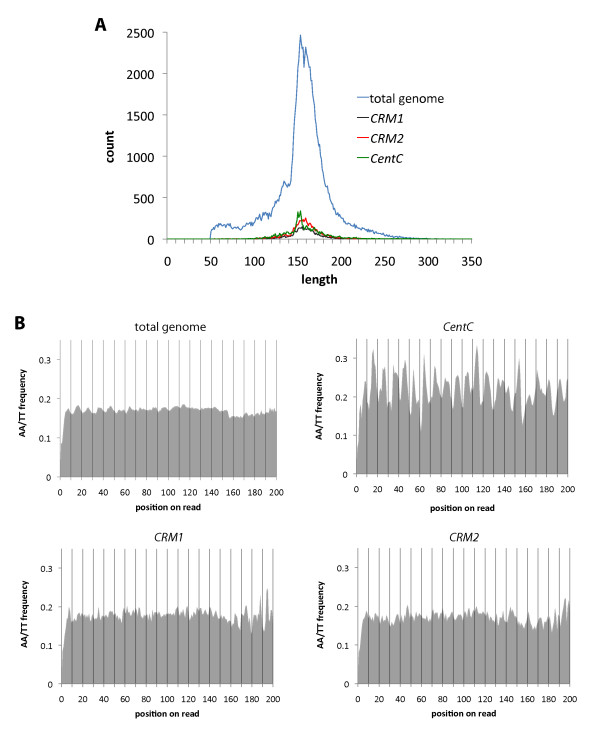
**CENH3 nucleosome core lengths and AA/TT contents**. (A) Number of reads per read length (bp). Genome-matching reads with unambiguous termini favour a length of around 155 bp. 'total genome' includes everything that aligned to the maize genome, version 1. The three centromere elements are shown separately. See also Additional File 1, part B. (B) The frequency of AA or TT dimers at each position along the read lengths. The reads were aligned at their starts and the number of AA or TT dimers counted at each position for each aggregated set. We arbitrarily cut off the analysis at position 200 in order to avoid statistical noise from the low number of reads with lengths greater than 200 bases. In the total genomic reads, the change in AA/TT content visible near position 155 suggests a subtle distinction in nucleotide content between the nucleosome core DNA and linker DNA. The *CentC*, *CRM1*, and *CRM2 *reads were identified by blastall alignments. See also Additional File 1, part C.

We examined the lengths and nucleotide composition of these reads and found two characteristics similar to those of general nucleosome cores: First, a predominant length of ~155 bp, slightly larger than the 147 bp typical of canonical H3 nucleosome core DNA in other organisms (Figure 1A; also see Additional File 1). Second, a tendency to favour either AA or TT dinucleotides every 10 bp. We calculated the number of AA or TT dimers at each position along the length of the aggregated, complete set of reads, aligned at their starts, and we observed a ~10-bp periodicity consistent with nucleosome structure and previous deep sequencing analyses [7,10] (Figure 1B; Additional File 1). Furthermore, in cases where the MNase products were substantially longer than an expected nucleosome core length, a subtle change in AA/TT content was visible (near bp 155; Figure 1B), providing further support for these reads being derived from nucleosome cores.

### AA/TT periodicity is a prominent feature of nucleosome positioning on *CentC *arrays, but not on *CRM1 *and *CRM2 *retrotransposons

The tendency for AA/TT periodicity in the underlying DNA indicates that centromere DNA sequence contributes to centromere nucleosome positioning. In order to further examine this, we compared results for nucleosomes from different centromere DNA elements. The three most substantial centromere components identified in maize DNA are the ~156-bp *CentC *elements which exists in long tandem arrays, the ~7.5-kbp retrotransposon *CRM2*, and the ~7-kbp retrotransposon *CRM1 *[31-34]. All three of these are highly enriched for centromere core regions and are conserved in other cereals. We aligned the total genomic reads to these elements and measured read counts, dinucleotide periodicities, and nucleosome position and spacing preferences. With our alignment parameters, 7.6% of the reads corresponded to *CentC*, 7.0% to *CRM2 *and 4.2% to *CRM1 *(Figure 1A; Additional File 1). While neither *CRM1 *nor *CRM2 *reads showed detectable AA/TT periodicities, *CentC *reads showed a dramatic ~10-bp AA/TT periodicity (Figure 1B). A control dataset of 454 reads unenriched for CENH3 and which was not subjected to micrococcal nuclease (MNase) digest lacked this periodicity, which indicated that it was not an artefact of library preparation or sequencing technology (Additional File 1).

### Distinct nucleosome phasing constraints on *CRM2 *and *CRM1 *retrotransposons

**Figure 2:**
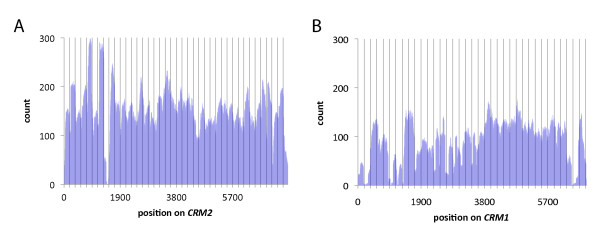
**Alignments of CENH3-ChIP reads to *CRM1 *and *CRM2***. Number of CENH3-ChIP reads aligning to each position on *CRM2 *(A) and *CRM1 (B)*. Vertical lines are spaced every 190 bp. In the case of *CRM2*, but not *CRM1*, alignments show strong tendency to peak every 190 bp, particularly toward its long terminal repeats (LTRs), indicating nucleosome phasing.

**Figure 3:**
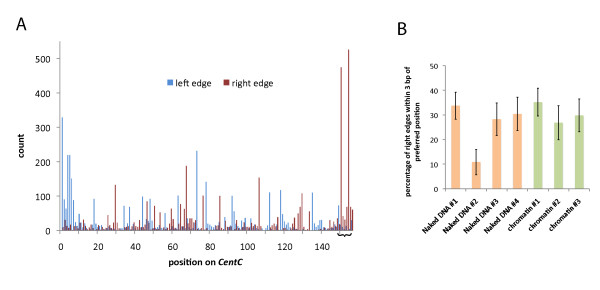
***CentC *alignments and micrococcal nuclease (MNase) sequence preference**. (A) The number of alignment edges at each position on *CentC *from CENH3-ChIP reads. Due to the short and repetitive character of *Cent*, just the alignment edges are depicted rather than the entire alignments. An apparent preferred position is defined by right edges positioned over the bracketed section (but see B). Alignments to *CentC *were split into two categories, forward and reverse orientation; then the 5'-most position of the forward alignments were combined with the 3'-most position of the reverse alignments to count the total number of left edge positions along the length of *CentC*. Likewise, the 5'-most position of the reverse alignments and 3'-most position of the forward alignments were combined to count the total number of right edge positions. Since this analysis uses only information from alignment edges, the alignments were first filtered so as to include only the ones with approximate nucleosome lengths (145 to 175 bp). (B) Sequence preference in MNase digestion of *CentC*. DNA fragments produced by MNase digest of naked DNA or chromatin were captured by ligation to adapters then amplified with primers that selected for *CentC*-adapter junctions (corresponding to the right edges shown in A). The bar chart indicates the number of cloned fragments (per 100) whose right edges were within 3 bp of the enriched site found in the CENH3-ChIP 454 dataset (the bracketed section in A, centred on the last base in GGGTGTCGGGGTG). Errors bars are standard errors of the means, calculated based on the sample sizes (74 sequences for 'Naked DNA No.1', 37 for 'Naked DNA No.2', 46 for 'Naked DNA No.3', 46 for 'Naked DNA No.4', 71 for 'Chromatin No.1', 41 for 'Chromatin No.2' and 47 for 'Chromatin No.3'.

Visual analysis of the alignments for each element revealed strong constraints on nucleosome positioning, or phasing, on *CRM2*, especially in its highly conserved long terminal repeat (LTR) regions [33] (Figure 2A). In particular, peaks in nucleosome occupancy occurred on ~190-bp intervals. Alignment positions on *CRM1*, in contrast, showed very little constraint, suggesting a lack of nucleosome phasing (Figure 2B). In the case of *CentC*, the alignments showed a diversity of allowed positions but with an enrichment for a single position at the ends of the sequence shown in Figure 3A. One concern is that inherent MNase cleavage biases could explain the enrichment for such a single position [35-37], since any site favoured by MNase on *CentC *would be repeated every 156 bp in long arrays. In order to test whether the favoured position on *CentC *is a result of nucleosome occupancy or MNase bias, we measured the relative enrichment of MNase fragments from both naked DNA and chromatin by selective amplification and Sanger sequencing. The results of this analysis clearly indicate that the enriched position can be explained as a consequence of MNase sequence preference (Figure 3B). It remains possible that an actual nucleosome position overlaps with a site of inherent MNase bias or that phasing could involve a complex relation with higher order *CentC *polymers. However, a one-nucleosome/one-repeat scenario would require unusually compact chromatin and would be hard to reconcile with the existence of even shorter tandem repeats of soybean (92 bp), mouse (120 bp) and sugarcane (140 bp).

### Uniform nucleosome spacing on *CentC *arrays and on *CRM1 *and *CRM2 *retrotransposons

**Figure 4:**
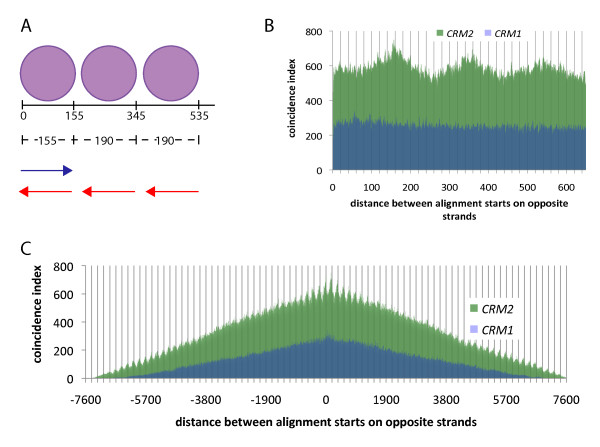
**Start-to-start distance analysis of nucleosome spacing on *CRM1 *and *CRM2***. (A) A schematic description of the start-to-start distance analysis. Three 155-bp nucleosomes (represented by purple circles) starting 190 bp apart give rise to 155-bp reads with alignments of both orientations--forward in blue, reverse in red. Distances between the start of the first forward alignment and start of the first reverse alignment correspond to the length of one nucleosome core. Distances between start of the first forward alignment and subsequent reverse alignments increase by 190 bp, corresponding to an additional nucleosome core and linker region. (B) A short range start-to-start distance analysis for *CRM1 *and *CRM2 *nucleosomes. The number of starts separated by each possible distance was quantified in terms of a coincidence index value. We arbitrarily cut off this plot at 650 bp to emphasize the presence of a peak at ~155 bases (as well as two subsequent peaks separated by ~190 bp). Vertical lines are spaced every 20 bp. (C) A long range start-to-start distance analysis for *CRM2 *CENH3 nucleosomes. Distances between start positions from alignments of opposite orientations were calculated and the number of starts separated by each possible distance between -7577 and +7577 bp quantified in terms of a coincidence index value. The length of the *CRM2 *reference sequence is 7577 bp, precluding any longer distances. For a random sampling of alignment pairs, the probability is lowest in order for them to be separated by the longest possible distance (the element's length), as there is only one possible combination for either the positive or negative extreme. In contrast, the number of combinations that allow for a distance of zero is almost as large as the length of the element. Hence, the triangular shape of the plot, with a peak centred near zero and tapering off in both directions. Vertical lines are spaced every 190 bp.

Spacing between nucleosomes is known to involve factors other than DNA sequence [38-40]. We wondered whether CENH3 nucleosomes would exist in arrays with regularly spaced nucleosomes across the whole centromere core or whether spacing patterns would vary within different centromere regions depending on the underlying DNA sequence. The alignments to *CRM2 *(Figure 2A) showed a tendency for spacing of nucleosomes on 190-bp intervals. In order to quantify this, we calculated distances between alignment start positions from each strand of DNA. As depicted in Figure 4A, a strongly positioned nucleosome would be predicted to produce start-to-start distances that match the size of one nucleosome core at roughly 155 bp. Over longer distances, we expect this distance to reflect the overall spacing of the nucleosomes including linkers. If we first align all CENH3 ChIP reads to the *CRM2 *consensus sequence and, beginning with a start site demarcating one end of a nucleosome core region, plot the distance to a start site on the opposite strand and the start site after that, and so on, we will reveal the spacing parameters typical of the *CRM2 *sequence. As can be seen in Figures 4B and 4C, these data show an unequivocal 190 base periodicity that spans the entire element (though less evident in the intermediate distances, consistent with the weaker positioning seen in the central, genic area of the retrotransposon; Figure 2A).

**Figure 5:**
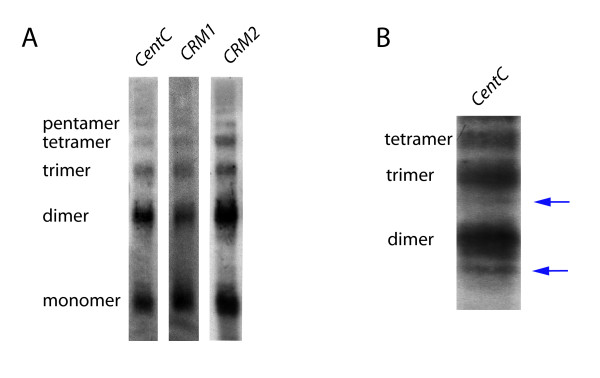
**A Southern analysis of nucleosome spacing on *CRM1*, *CRM2 *and *CentC***. Southern blots of micrococcal nuclease (MNase) digested CRM1, *CRM2 *and *CentC *nucleosome cores. (A) Total chromatin was lightly digested with MNase and isolated DNA fragments corresponding to nucleosome monomers, dimers and higher order polymers were separated by gel electrophoresis and probed for *CentC *and for *CRM1 *and *CRM2 *LTRs. (B) A rare fragment, ~30-bp shorter than the majority of the nucleosome dimers, was often visible, especially for *CentC *(blue arrow). In some cases a shorter fragment could also be seen in the trimer range.

In contrast to *CRM2*, *CRM1 *lacked both the single-nucleosome peak at start-to-start distance 155 as well as the subsequent peaks every 190 bp (Figure 4B and 4C), consistent with the lack of phasing seen in Figure 2B. This result does not rule out uniform spacing: Even a perfectly uniform spacing arrangement would be undetectable without phasing constraint. Similarly, since *CentC *is only long enough to accommodate a single nucleosome and longer arrays consist of highly similar repeats, we could not perform meaningful start-to-start distance analyses to measure nucleosome spacing. Instead, we used Southern blotting to compare nucleosome polymer lengths from MNase-treated chromatin with probes for *CentC*, *CRM1 *and *CRM2*. Intriguingly, the greater part of the signals from all three probes was indistinguishable and yielded DNA fragment sizes corresponding to ~190-bp nucleosome spacing (Figure 5A). Combined with the lack of measurable phasing on *CentC *(not attributable to MNase sequence preference), this result argues against any substantial level of one-nucleosome/one-repeat phasing on *CentC*. 190-bp spacing was also the predominant arrangement for nucleosome polymers genome-wide, as evidenced by ethidium bromide staining of total chromatin after MNase digest (Additional File 2).

A second, minor potential spacing pattern was also suggested, apparently derived from adjacent nucleosomes with short or absent linkers (Figure 5B). This was manifest as a ~30-bp shorter fragment in the nucleosome dimer range, particularly for *CentC*. In some cases we could also distinguish a shorter band in the nucleosome trimer range, but higher order polymers did not give sufficient resolution on the gel. (The shorter fragments did not appear to be centromere specific, as they were also present in arrays of the maize 180-bp *Knob *repeat; data not shown). Another piece of evidence for deviations from perfect, absolute 190-bp spacing is the 156-bp length of *CentC *and the tendency for nucleosome positions to conform to the underlying AA/TT distribution. Periodic deviations of a few bases would be required in order to retain the expected conformation between the histones and the DNA (though an average 190-bp spacing could be maintained).

Since the chromatin used for Southerns was not enriched for CENH3 by ChIP, a substantial portion of the signal from each element resulted from canonical H3 nucleosomes. Even the analyses from CENH3 ChIP cannot fully distinguish between canonical H3 and CENH3 effects if both types of histones coexist in nucleosomes arrays (which is unknown at this time, but would be consistent with the unusual timing of CENH3 deposition compared to canonical H3 deposition [41-43]). Nonetheless, these data suggest that the dominant theme for nucleosomes on *CRM1*, *CRM2 *and *CentC *elements (as well as the genome as a whole) is spacing intervals of ~190-bp on average - though the mode of positioning and rigidity of the chromatin depends on the local DNA.

## Conclusions

We conclude from these data that structural features of centromere chromatin can be linked to the underlying DNA and that this can result in non-uniform chromatin environments within CENH3 regions. In addition, factors independent of local DNA impose a generally similar nucleosome spacing arrangement. It has long been known that centromere chromatin is more conformationally rigid than other chromatin and this has been proposed to stem, in part, from intrinsic DNA bending/nucleosome interactions [12-15]. We further support this idea with our finding that nucleosome positioning on tandem repeats adapts to the underlying AA/TT distribution. The absence of a nucleosome phasing signal on the tandem repeat in maize contrasts with both the phasing reported on tandem repeats in primates and with the phasing on the maize retrotransposon *CRM2*. Interestingly, for *CRM2*, the dominant positioning frame is specified without a recognizable AA/TT pattern. However, a strong sequence motif may be present and through enforcing the exact position of one nucleosome on *CRM2*, could set the phase for the entire array of nucleosomes. Such a motif need not interact directly with the nucleosome. For example, binding of the transcription factor CTCF sets the positions of large nucleosome arrays [44]. The retrotransposon *CRM1 *presents yet a different situation, with neither a detectable correlation with AA/TT dimers nor any strongly favoured positioning frame.

Centromere DNA presents a puzzle in that although it tends to have less conservation than the genome as whole and is largely made up of unstable repetitive elements, it forms the basis for a highly conserved and essential structure. If the DNA makeup were important for kinetochore function, one would expect to find mechanisms for preserving centromere DNA integrity, mechanisms for adapting to a fluid DNA landscape, or both [45]. Whatever the forces in play, our data suggest that centromere sequences are limited by constraints on AA/TT periodicity and other features affecting chromatin structure.

An important consideration in interpreting these results is the structure of CENH3-containing nucleosomes. Recent studies of animal centromeres suggest that CENH3 exists in a specialized smaller nucleosome made up of a tetramer of H2A, H2B, H4 and CENH3 with the DNA wrapped around the nucleosome in a right-handed configuration rather than the canonical histone octamer with the DNA wrapped around in a left-handed configuration [46-48]. In contrast, other studies have shown that CENH3 is capable of forming octameric nucleosomes, and a recent DNA topological analysis revealed DNA in a left-handed configuration [49]. Consequently, a lively debate is taking place as to the relative importance of both nucleosome structures (and other alternative ones). Since the data supporting the presence of tetrameric nucleosomes in animals is compelling and CENH3 is highly conserved, we expect that functionally important tetramers will also be found in maize. As such, we might have also expected to see a substantial amount of smaller nucleosome core particles corresponding to tetrameric nucleosomes in the 454 sequencing dataset and Southern blots (Figures 1A and 4). Nevertheless, we did not see such structures despite the fact that the sample was highly enriched for the genetically-defined centromeric regions and were not only enriched for *CentC *(33.5 fold enrichment), *CRM1 *(16.3 fold) and *CRM2 *(51.1 fold) but for hundreds of other unique sites within the centromere core regions [29]. In fact, these reads were used to precisely define centromere cores [29,30]. Therefore, our data suggest that the 155-bp, CENH3-containing nucleosomes are a major component of the functional centromere chromatin. It remains possible that specificities of the antibodies or the extent of MNase digestion may have biased the analysis towards octameric structures. Perhaps more important is the fact that most of the cells in young leaves have ceased division and are, presumably, at the G1 phase of the cell cycle when CENH3 nucleosomes are stable and fully assembled. One way to explain the inconsistencies between our data and prior work in animals would be to postulate that tetrameric nucleosomes are more common in dividing cells as an assembly intermediate following DNA replication but before CENH3 deposition.

In summary, these results indicate that centromere DNA elements influence chromatin structure but leave open the question of whether one or more of these distinct modes of nucleosome positioning have consequences in terms of centromere specification and kinetochore function. The identification of apparently stable neocentromeres that lack canonical centromere elements suggests limits to the importance of centromere DNA sequence (for recent review, see [50]). However, in maize, after a centromere has been epigenetically deactivated (when CENH3 and other kinetochore markers are lost), the same region can regain activity, hinting that centromere elements present a particularly permissive environment for centromere formation [51]. Similarly, satellite DNA is known to be important for human artificial chromosomes [2,52] and the non-standard geometry of neocentromeres is associated with kinetochore defects [53]. These data, and the fact that kinetochore positions are remarkably stable over long periods of time, make a strong argument for the functional consequences of centromere DNA. Our data suggest that this favourable environment in maize includes multiple DNA elements including *CRM2 *- which can strongly phase CENH3 nucleosomes - and *CentC*, whose sequence makeup readily conforms to the tight bending of a nucleosome core structure.

## Methods

### 454 read processing and aligning

The 454 outputs in SFF format were first converted to fasta format using sff_extract [http://bioinf.comav.upv.es/sff_extract/]. The reads were then scanned from the 3' end for the first instance of "CTGAGACAC" (the first nine bases of the 454 adapter) and trimmed back to that point. Reads that lacked this sequence or were shorter than 39 bases were discarded. The resulting set of trimmed reads was aligned to the maize genome, version 1, using a local installation of BLAT [54]. The following parameters were changed from default settings: -fastMap,

-minIdentity = 99, -maxIntron = 5, and -minScore = 30 (87% of the CENH3 reads and 82% of the control reads could be aligned with the much more stringent setting

-minScore = 300). The resulting reads with genomic matches were then aligned to a reference set of DNA sequences consisting of a *CentC *trimer, a *Knob180 *trimer and *CRM1*-*CRM4*. For this much smaller reference, a local installation of blastall was used, with default DNA parameters except that expectation value (-e) was set to 1 × 10^-15^. In cases where a single read produced multiple equally good alignments, only one alignment was kept.

### Start-to-start distance analysis

Alignments were separated based on orientation relative to the reference sequence and the distance between forward- and reverse-oriented alignment start positions calculated using a similar method as Gent *et al. *[55]. In brief, this analysis quantified the tendency for alignment start positions, 'starts', to occur at particular distances from each other, 'start-to-start distances'. The number of starts at each position was counted and then the distances between all possible pairwise combinations of starts were measured and assigned a 'coincidence index' value related to the cube root of the number of starts separated by that distance.

### Control for MNase bias in digestion of *CentC*

#### Chromatin isolation

Whole maize seedlings were flash frozen in liquid nitrogen and ground to a powder with a prechilled mortar and pestle; 10 mL ground tissue was homogenized by vigorous shaking in a 50 mL volume of TBS (Tris-buffered saline) plus 0.5% Tween 40, filtered through two successive layers of 35-μm-pore Nitex bolting cloth (Wildlife Supply Co, FL, USA), then the nuclei pelleted by centrifugation at 600 g for 10 min. After removing the supernatant, the nuclei were gently suspended in 5 mL of 25% sucrose in TBS, overlaid onto 2.5 mL of 50% sucrose in TBS, then centrifuged at 1500 g for 20 min. At all times, including during centrifugation, the material was kept near 4°C.

#### Micrococcal nuclease digestion

Isolated chromatin and naked DNA samples were suspended in digestion buffer (0.32 M sucrose, 50 mM Tris, 4 mM MgCl_2_, 1 mM CaCl_2_). MNase (2 × 10^6 ^gel units/mL; New England Biolabs, MA, USA, No. M0247S) was added at about 1 μL per 500 μL of digestion buffer solution (on ice). The MNase and DNA mixtures were then separated into several smaller volumes for incubations at 37°C for different time periods (30 s to 3 min). The reactions were stopped and the DNA purified as described previously [7].

#### Sequencing library preparation

The purified DNA fragments were run on 3.5% NuSieve^® ^GTG^® ^Agarose (Lonza, Basel, Switzerland) gels, and the nucleosome monomer size range, 140-190 bases, excised and extracted from the gels with a QIAquick^® ^Gel Extraction Kit (QIAGEN Sciences, Maryland, USA). The samples were kept at room temperature during the entire procedure to avoid denaturation of the complex pools of DNA fragments. The fragments were then inserted between 5' and 3' adapters and amplified using a method similar to that of Gu and Fire [56]. A key difference in our method was that, rather than using the published primers that bind to both the 5' and 3' adapters and amplify independently of the DNA insert sequence, we selected for *CentC *inserts with a *CentC*-specific primer. The primer sequences were: GTGGTTTCGCGCAATTTCGTTGTC (KD-JIG-72, binds *CentC*) and AAGACGGCATACGAGCTCTTCCGAT (KD-JIG-98, binds the 3' adapter). Hence, the junctions of the 3' adapter and 5' portions of *CentC *inserts were amplified selectively. Amplified products of ~30 to 200 bp were again gel purified (3.5% NuSieve agarose) and extracted with a QIAquick kit without heating, then cloned and Sanger sequenced.

### Southern blotting

In general, all steps were as described in the *DIG Application Manual for Filter Hybridization *by Roche (Basel, Switzerland) http://www.roche-applied-science.com/PROD_INF/MANUALS/DIG_MAN/dig_toc.htm; specific details were as follows: purified MNase digested DNA fragments were produced as described above and were then separated on a 4% NuSeive agarose gel for at least 3 h at 100 V and then transferred to a Amersham HyBond-N^+ ^(GE Healthcare, NJ, USA) membrane by capillary action overnight without prior depurination of the DNA. Membranes were baked at 80°C for 2 h after transfer. Probes were allowed to hybridize overnight in DIG Easy Hyb (Roche No. 11 603 558 001) at 42°C for *CRM1 *and *CRM2 *and 44°C for *CentC*. All three probes were ~150 bases in length and were produced with the PCR DIG Probe Synthesis Kit (Roche No. 11 636 090 910). Primers were as follows: For *CentC*, GCCACCGGAACCATTTCTTCGTTT (KD-JIG-63) and TCGTGCTTTGTATGCACCC (KD-JIG-91); for *CRM1*, ATCTCCACTCACCGAAAGATTGGG (KD-JIG-82) and AAGGGTGCTGGGATAAGGTCTAAC (KD-JIG-83); and for *CRM2*, TTGTAAGCGCGCGTGCTAGTTCA (KD-JIG-86) and ACTCGTCCTGCAAGCAATCGAAGA (KD-JIG-87). For C*RM1 *and *CRM2 *detection, the high stringency buffer was 0.5% SDS (sodium dodecyl sulphate) at a temperature of 65°C; for *CentC *it was 0.1% SDS at 68°C. Detection was carried out with the DIG Luminescent Kit for Nucleic Acids (Roche No. 11 363 514 910)

### Preparation of a control 454 library

Total chromatin was extracted from maize (inbred B73 stock) immature ears, chemically crosslinked and fragmented by sonication [57]. After an, at best, marginally successful attempt at enrichment for centromeric sequences by ChIP (immunoprecipitation with an antibody against maize Centromere Protein C), the DNA was processed and 454 sequenced.

## Abbreviations

ChIP: chromatin immunoprecipitation; CENH3: centromeric histone H3; CENP: centromere protein; MNase: micrococcal nuclease; LTR: long terminal repeat.

## Competing interests

The authors declare that they have no competing interests.

## Authors' contributions

JIG, CNT and CR performed the experiments. JIG, KLS and GGP performed the bioinformatic analyses. RKD, JIG and GGP designed the study. JIG and RKD wrote the manuscript. All authors read and approved the final manuscript.

## Supplementary Material

Additional file 1 **454 control reads for centromere enrichment, length distribution and AA/TT content**. (A) Enrichment of reads for centromere DNA. Genome-matching, CENH3-chromatin immunoprecipitation (ChIP) and control reads were aligned to a set of reference DNA sequences consisting of *CentC*, *CRM1*, *CRM2*, *CRM3*, *CRM4 *and *Knob180 *by blastall. The number of reads that map to each alignment relative to the number of reads that map to the genome is shown. In order to allow for the complete alignment of any theoretical perfect matching read up to 313 bp, a trimer of *CentC *was used rather than a single unit. *Knob180 *was also trimerized. In cases where a single read produced an alignment to multiple elements, only the longest alignment was counted. While *Knob180 *is an extremely abundant repeat, it is not a component of centromeres; neither have we observed CENH3 localized to *knob *repeats by immunolocalization. We do not know the reason for the strong *knob *signal in the CENH3 reads but suspect some level of background non-CENH3 nucleosome cross-reactivity with the antibody [30]. The control reads provide a comparison for the potential biases introduced during 454 sequencing or library preparation. (B) The number of control reads per read length (bp). Genome-matching reads with unambiguous termini do not strongly favour a particular length. (C) The frequency of AA or TT dimers at each position in first 200 bp of each control read. The reads were aligned at their starts and the number of AA or TT dimers counted at each position for each aggregated set. We arbitrarily cut off the analysis at position 200 in order to avoid statistical noise from the low number of reads with lengths greater than 200 bp. In addition to a lack of detectable AA/TT periodicity, no change in AA/TT content is visible near position 155, in contrast with the CENH3 ChIP reads (see Figure 1). Click here for file

Additional file 2 **General genomic nucleosome spacing**. (A) A plot of gel migration versus DNA fragment length. Total chromatin was lightly digested with micrococcal nuclease (MNase) and run on a 4% NuSieve agarose gel along with a 100 bp DNA ladder. The known lengths of the ladder DNA were used to plot a standard curve of DNA length as a function of migration distance (dashed line and diamonds). The gel migration of each discernable band produced in the MNase digest, corresponding to successively larger polymers of nucleosome cores, was then mapped onto the standard curve to estimate its length. (B) Summary table of nucleosome spacing. Since a polymer of N nucleosomes contains N-1 linkers, the spacing between the start of each nucleosome in a nucleosome core N-mer should be (total length + one linker length)/N. Using a linker length of 35 bp gives a consistent nucleosome spacing distance of ~190 bp for the polymers examined (dimers to hexamers). The monomer length of ~175 bp is larger than expected due to the incompleteness of the digest (required for retention of larger polymers). Click here for file
