# Supplementary figures and images for: Distinct influences of tandem repeats and retrotransposons on CENH3 nucleosome positioning
Source: Epigenetics Chromatin. 2011 Feb 25;4:3. doi: 10.1186/1756-8935-4-3 (PMC3053214; doi:10.1186/1756-8935-4-3)

Control read centromere enrichment, length distribution, and AA/TT content

**A**

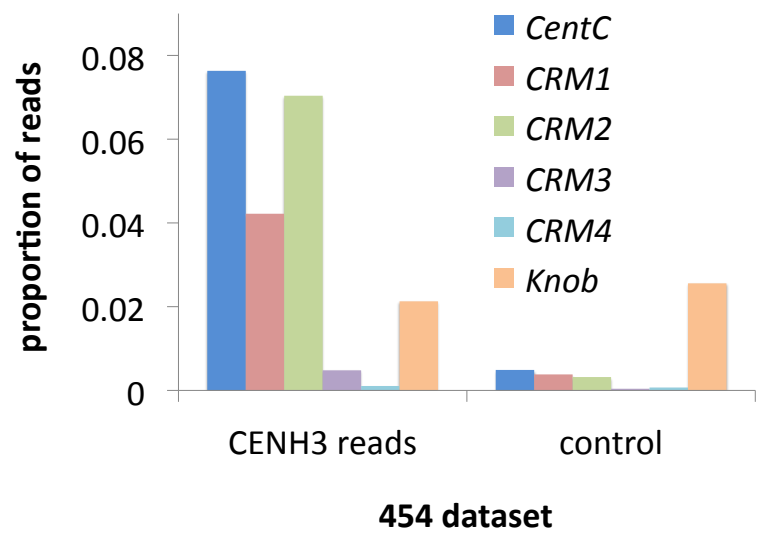

**B**

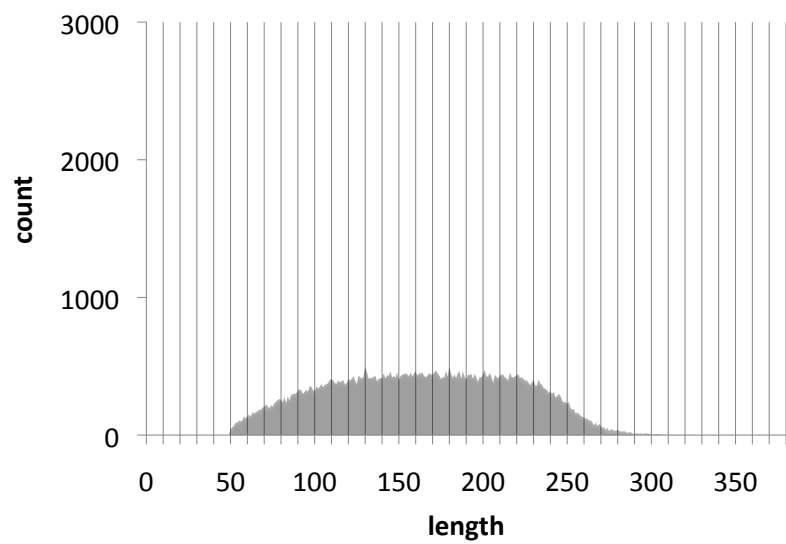

**C**

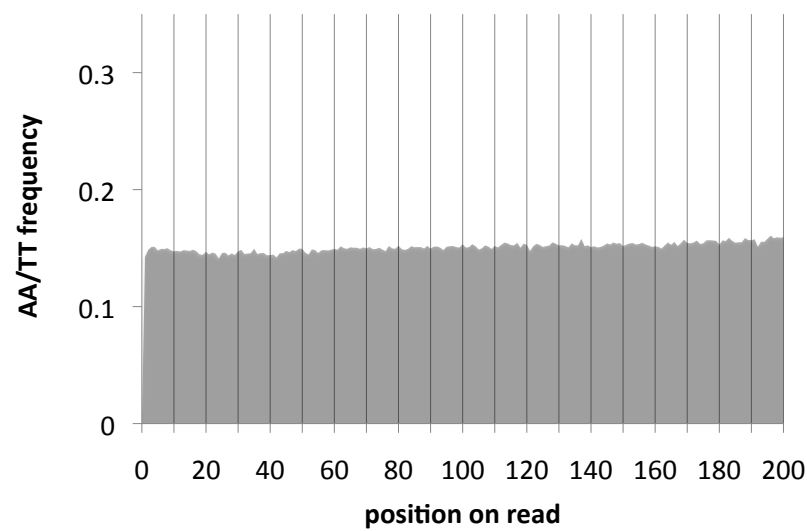

Supplement: Additional file 1 — 454 control reads for centromere enrichment, length distribution and AA/TT content. (A) Enrichment of reads for centromere DNA. Genome-matching, CENH3-chromatin immunoprecipitation (ChIP) and control reads were aligned to a set of reference DNA sequences consisting of CentC, CRM1, CRM2, CRM3, CRM4 and Knob180 by blastall. The number of reads that map to each alignment relative to the number of reads that map to the genome is shown. In order to allow for the complete alignment of any theoretical perfect matching read up to 313 bp, a trimer of CentC was used rather than a single unit. Knob180 was also trimerized. In cases where a single read produced an alignment to multiple elements, only the longest alignment was counted. While Knob180 is an extremely abundant repeat, it is not a component of centromeres; neither have we observed CENH3 localized to knob repeats by immunolocalization. We do not know the reason for the strong knob signal in the CENH3 reads but suspect some level of background non-CENH3 nucleosome cross-reactivity with the antibody [30]. The control reads provide a comparison for the potential biases introduced during 454 sequencing or library preparation. (B) The number of control reads per read length (bp). Genome-matching reads with unambiguous termini do not strongly favour a particular length. (C) The frequency of AA or TT dimers at each position in first 200 bp of each control read. The reads were aligned at their starts and the number of AA or TT dimers counted at each position for each aggregated set. We arbitrarily cut off the analysis at position 200 in order to avoid statistical noise from the low number of reads with lengths greater than 200 bp. In addition to a lack of detectable AA/TT periodicity, no change in AA/TT content is visible near position 155, in contrast with the CENH3 ChIP reads (see Figure 1). [file 1756-8935-4-3-S1.PDF]
