# Supplementary material for: Distinct influences of tandem repeats and retrotransposons on CENH3 nucleosome positioning
Source: Epigenetics Chromatin. 2011 Feb 25;4:3. doi: 10.1186/1756-8935-4-3 (PMC3053214; doi:10.1186/1756-8935-4-3)
Supplement: Additional file 2 — General genomic nucleosome spacing. (A) A plot of gel migration versus DNA fragment length. Total chromatin was lightly digested with micrococcal nuclease (MNase) and run on a 4% NuSieve agarose gel along with a 100 bp DNA ladder. The known lengths of the ladder DNA were used to plot a standard curve of DNA length as a function of migration distance (dashed line and diamonds). The gel migration of each discernable band produced in the MNase digest, corresponding to successively larger polymers of nucleosome cores, was then mapped onto the standard curve to estimate its length. (B) Summary table of nucleosome spacing. Since a polymer of N nucleosomes contains N-1 linkers, the spacing between the start of each nucleosome in a nucleosome core N-mer should be (total length + one linker length)/N. Using a linker length of 35 bp gives a consistent nucleosome spacing distance of ~190 bp for the polymers examined (dimers to hexamers). The monomer length of ~175 bp is larger than expected due to the incompleteness of the digest (required for retention of larger polymers). [file 1756-8935-4-3-S2.PDF]

Spacing between total genomic nucleosomes

A

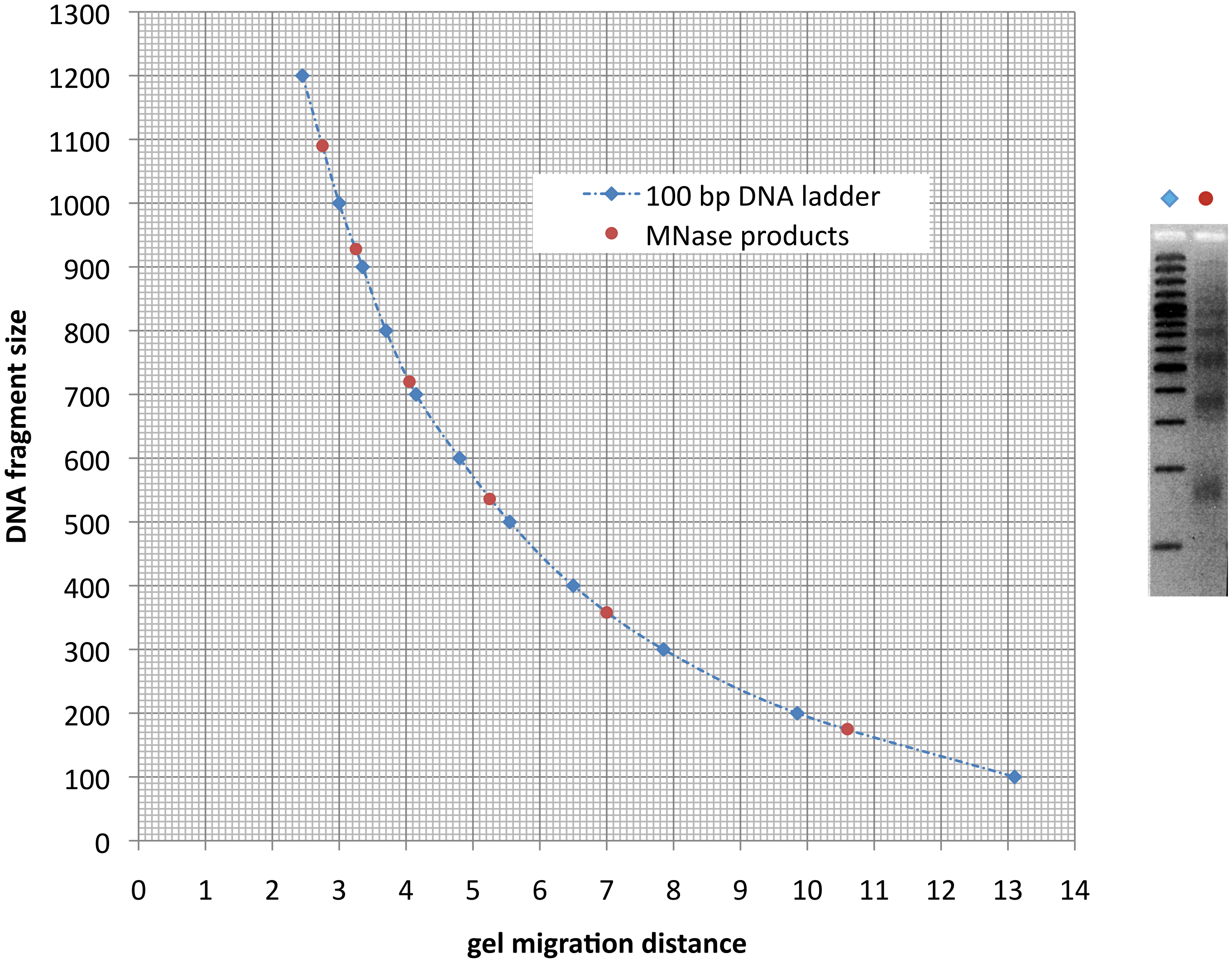

B

Nucleosome core polymer	Total length (bp)	Nucleosome spacing (bp)
hexamer	1090	186
pentamer	928	193
tetramer	720	189
trimer	536	190
dimer	358	197
monomer	175	N/A
average		191
standard deviation		3.6
